# Acidosis Decreases c-Myc Oncogene Expression in Human Lymphoma Cells: A Role for the Proton-Sensing G Protein-Coupled Receptor TDAG8

**DOI:** 10.3390/ijms141020236

**Published:** 2013-10-11

**Authors:** Zhigang Li, Lixue Dong, Eric Dean, Li V. Yang

**Affiliations:** 1 Department of Oncology, Brody School of Medicine, East Carolina University, Greenville, NC 27834, USA; E-Mails: liz@ecu.edu (Z.L.); dongl@ecu.edu (L.D.); ehdean@live.unc.edu (E.D.); 2 Department of Internal Medicine, Brody School of Medicine, East Carolina University, Greenville, NC 27834, USA; 3 Department of Anatomy and Cell Biology, Brody School of Medicine, East Carolina University, Greenville, NC 27834, USA; 4 Lineberger Comprehensive Cancer Center, UNC at Chapel Hill, NC 27599, USA

**Keywords:** acidosis, tumor microenvironment, TDAG8, GPR65, c-Myc

## Abstract

Acidosis is a biochemical hallmark of the tumor microenvironment. Here, we report that acute acidosis decreases c-Myc oncogene expression in U937 human lymphoma cells. The level of c-Myc transcripts, but not mRNA or protein stability, contributes to c-Myc protein reduction under acidosis. The pH-sensing receptor TDAG8 (GPR65) is involved in acidosis-induced c-Myc downregulation. TDAG8 is expressed in U937 lymphoma cells, and the overexpression or knockdown of TDAG8 further decreases or partially rescues c-Myc expression, respectively. Acidic pH alone is insufficient to reduce c-Myc expression, as it does not decrease c-Myc in H1299 lung cancer cells expressing very low levels of pH-sensing G protein-coupled receptors (GPCRs). Instead, c-Myc is slightly increased by acidosis in H1299 cells, but this increase is completely inhibited by ectopic overexpression of TDAG8. Interestingly, TDAG8 expression is decreased by more than 50% in human lymphoma samples in comparison to non-tumorous lymph nodes and spleens, suggesting a potential tumor suppressor function of TDAG8 in lymphoma. Collectively, our results identify a novel mechanism of c-Myc regulation by acidosis in the tumor microenvironment and indicate that modulation of TDAG8 and related pH-sensing receptor pathways may be exploited as a new approach to inhibit Myc expression.

## 1. Introduction

The c-Myc oncogene is a member of the Myc family, which also includes l-Myc, *N*-Myc, *S*-Myc and B-Myc [1–3]. Myc plays critical roles in many aspects of cellular processes, such as cell growth, metabolism, apoptosis and cell fate determination [4,5]. c-Myc promotes cell cycle progression from the G0/G1 into the S phase by activating cell cycle-promoting genes and inhibiting cell cycle suppressors [6–8]. Furthermore, c-Myc regulates cell metabolism, synthesis of RNA and protein, as well as genomic instability and metastasis [9–13]. c-Myc, synergizing with other oncogenes, such as BCL-2, RAS and RAF, drives cell transformation [14]. Myc deregulation is associated with tumorigenesis and poor disease prognosis. Aberrant overexpression of Myc through gene amplification and translocation and transcriptional and translational regulations has been observed in many types of cancer [1].

The expression of c-Myc is tightly regulated at multiple levels [1,5,15–17]. At the transcriptional level, the c-Myc promoter is potentially bound by several transcriptional factors, such as TCF4, LEF-1, β-catenin, γ-Catenin, E2F-1, p53 and NFκB. Many signaling pathways, including the PI3K-Akt-mTOR, Ras-Raf-MEK-ERK/MAPK, TGF-β, NFκB and Wnt pathways, regulate c-Myc mRNA expression. Small GTPase pathways, such as Ras and Rac, are also involved in c-Myc transcriptional regulation. Moreover, c-Myc expression is regulated at the posttranscriptional level, including mRNA stability and translation and protein stability and modification.

Tumor microenvironment is characterized by acidosis, which is due to the production of hydrogen ions from lactic acid by glycolysis (“Warburg effect”) and from other sources, such as carbonic acid and ATP hydrolysis [18–21]. Acidosis can be cytotoxic, retard cancer cell proliferation, induce dormancy and lead to apoptosis [22–26]. Microarray and biochemical studies demonstrate that acidosis inhibits glycolysis and Akt activities in breast cancer cells, and the gene signature induced by acute acidosis is correlated with favorable prognosis in breast cancer patients [27]. However, chronic acidosis is proposed as a selection pressure for cancer cell somatic evolution in a Darwinian manner [28–30]. Autophagy is a protective mechanism for tumor cells to survive in chronic acidosis [31,32]. It is also shown that acidosis facilitates cancer cell invasion by degrading extracellular matrix [33,34]. Thus, the roles of acidosis in tumor biology are very complex, depending on acute *versus* chronic effects and the biological context.

A family of G protein-coupled receptors (GPCRs), including GPR4, TDAG8 (GPR65), OGR1 (GPR68) and G2A (GPR132), has been identified as proton sensors [35–42]. TDAG8 is highly expressed in lymphoid tissues and lymphoma and leukemia cell lines [43–47]. Both tumor-promoting and tumor-suppressing activities of TDAG8 have been reported. Ectopic overexpression of TDAG8 increases the tumor growth of lung carcinoma cells [48]. In WEHI7.2 and CEM-C7 T-cell lymphoma cell lines, TDAG8 is activated by acidosis to promote the evasion of apoptosis under glutamine starvation [49]. On the other hand, TDAG8 has also been reported as a tumor suppressor, which promotes glucocorticoid-induced apoptosis in murine lymphoma cells and thymocytes [45,47]. Moreover, TDAG8 (T-cell death-associated gene 8) was originally identified as a gene substantially upregulated during T-cell apoptosis [43]. TDAG8 as a pH sensor is highly relevant in lymphomas, because these tumors have abundant proton production and high TDAG8 expression. However, the biological roles of TDAG8 in lymphoma remain ill-defined.

Here, we demonstrate that acidosis and TDAG8 suppresses the expression of the c-Myc oncogene in lymphoma cells. Our results also show that TDAG8 expression is significantly decreased in human lymphoma samples in comparison to normal lymphoid tissues, suggesting a potential tumor suppressor function of TDAG8 in lymphoma.

## 2. Results

### 2.1. c-Myc Protein Is Downregulated by Acidic pH Treatment in U937 Lymphoma Cells

**Figure 1:**
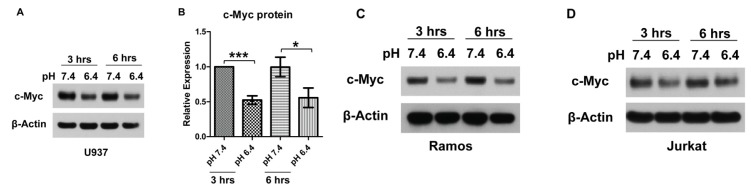
c-Myc protein is downregulated by acidosis in lymphoma and leukemia cell lines. (**A**) U937 lymphoma cells treated with pH 7.4 or pH 6.4 for three and 6 h were subject to Western blot assay using anti-c-Myc antibody. β-Actin was used as a loading control; (**B**) Quantification of U937 cell Western blot results. The expression of c-Myc in the 3-h pH 7.4 treatment was set as one. The data presented were derived from nine biological replicates. Error bars indicate ±SEM. *p < 0.05; ***p < 0.001; (**C**) Western blot analysis of Ramos lymphoma cells treated with pH 7.4 or pH 6.4 for three and 6 h; (**D**) Western blot analysis of Jurkat T-cell leukemia cells treated with pH 7.4 or pH 6.4 for three and 6 h.

The expression of the critical cell regulator c-Myc was examined in U937 lymphoma cells treated with the physiological pH 7.4 and the acidic pH 6.4. Western blotting with a c-Myc-specific antibody revealed that the c-Myc protein level was reduced by approximately 50% under the 3-h and 6-h treatment of pH 6.4 when compared to the pH 7.4 treatment (Figure 1A,B). Similar c-Myc downregulation by acidic pH was also observed in Ramos lymphoma cells and Jurkat T-cell leukemia cells (Figure 1C,D).

### 2.2. Downregulation of c-Myc Protein by Acidosis Is Due to Reduced c-Myc Transcriptional Level, but not mRNA or Protein Stability, in U937 Lymphoma Cells

**Figure 2:**
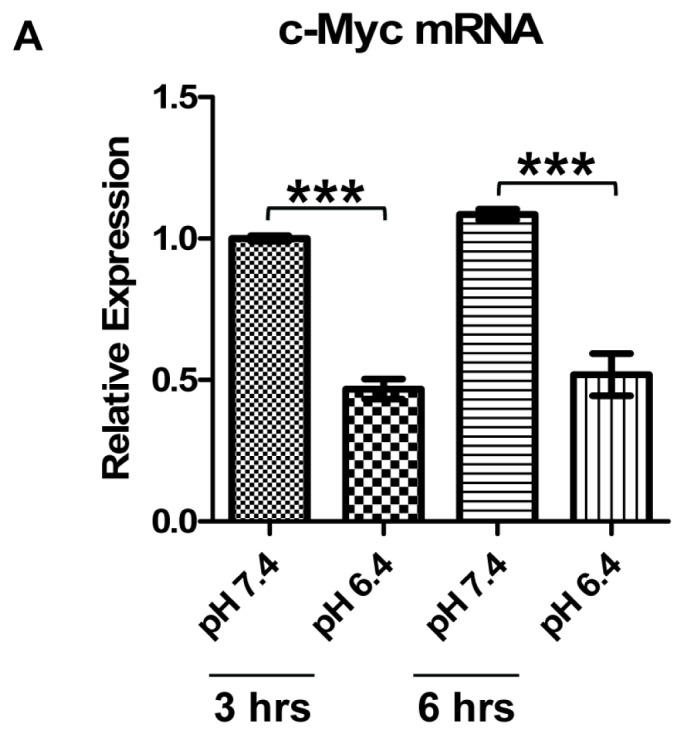

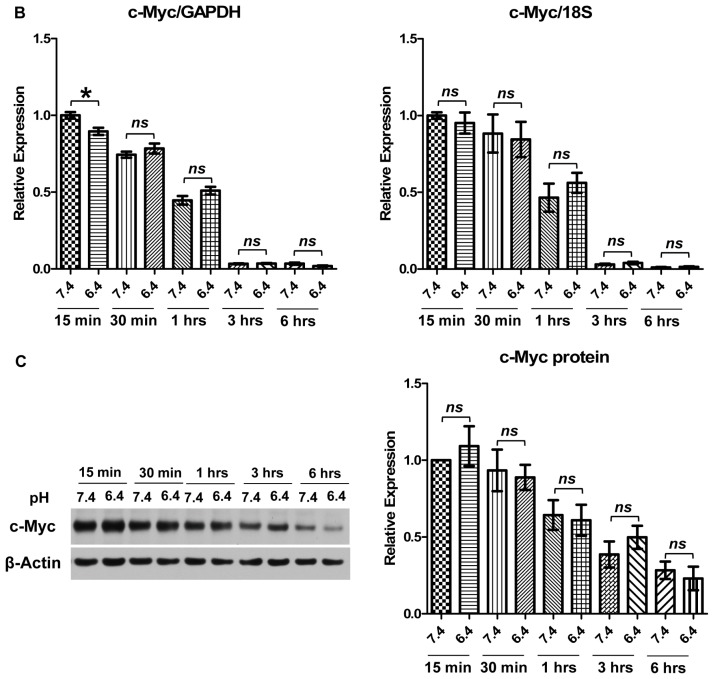
c-Myc transcriptional level, but not mRNA or protein stability, is downregulated by acidosis in U937 cells. (**A**) c-Myc mRNA is decreased by pH 6.4 treatment. U937 cells treated with pH 7.4 or pH 6.4 for three and 6 h were subject to total RNA isolation and real-time RT-PCR using c-Myc-specific primer probes. GAPDH (glyceraldehyde 3-phosphate dehydrogenase) was used as an internal control for normalization; (**B**) c-Myc mRNA stability is not altered by acidosis. U937 cells grown in HEM (7.5 mM HEPES, 7.5 mM EPPS and 7.5 mM MES)-buffered medium with 5 μg/mL actinomycin D at pH 7.4 or pH 6.4 for the indicated times were subject to total RNA extraction and real-time RT-PCR using c-Myc-specific primer probes. GAPDH (**left**) and 18S rRNA (**right**) were used as internal controls. The expression of c-Myc under 15 min of pH 7.4 plus actinomycin D treatment was set as one; (**C**) c-Myc protein stability is not altered by acidosis. **Left** panel: U937 cells grown in HEM-buffered medium with 50 μg/mL cycloheximide (CHX) at pH 7.4 or pH 6.4 for the indicated times were subject to total protein extraction and Western blot using anti-c-Myc antibody. β-Actin was used as a loading control. **Right** panel: quantification of Western blot results. The expression of c-Myc under the 15-min treatment with pH 7.4 and CHX was set as one. The data presented were derived from at least three biological replicates. Error bars indicate ±SEM. *p < 0.05; ***p < 0.001; ns, not significant (*p* > 0.05).

In order to elucidate the cause of c-Myc downregulation by acidic pH, the mRNA transcript level and the mRNA and protein stability of c-Myc were examined. Real-time RT-PCR (reverse transcriptase-polymerase chain reaction) showed that c-Myc mRNA was reduced by 50% under 3-h and 6-h pH 6.4 treatment (Figure 2A), which was close to the level of c-Myc protein reduction (Figure 1). The stability of c-Myc mRNA was determined by treating U937 cells with the transcription inhibitor actinomycin D, and then, the rate of c-Myc mRNA decay was measured by real-time RT-PCR. The half-life of c-Myc mRNA was approximately 1 h in U937 cells, and there was no significant difference in c-Myc mRNA stability between the treatment with pH 7.4 and pH 6.4, except a slight reduction of c-Myc/GAPDH, but not c-Myc/18S rRNA by pH 6.4 at 15 min (Figure 2B). To examine c-Myc protein stability, U937 cells were treated with the translation inhibitor cycloheximide and the c-Myc protein level was determined by Western blotting. The results showed that the half-life of c-Myc protein was approximately 3 h in U937 cells, and there was no significant difference in c-Myc protein stability between the pH 7.4 and the pH 6.4 treatments (Figure 2C). Altogether, our data suggest that mRNA expression level, but not mRNA or protein stability accounts for c-Myc downregulation under acidosis.

### 2.3. c-Myc Protein Is Decreased by TDAG8 Overexpression and Increased by TDAG8 Knockdown in U937 Lymphoma Cells

**Figure 3:**
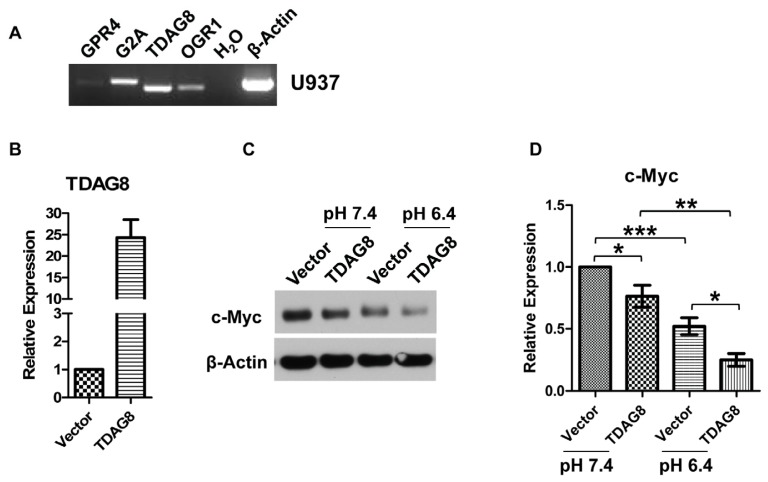

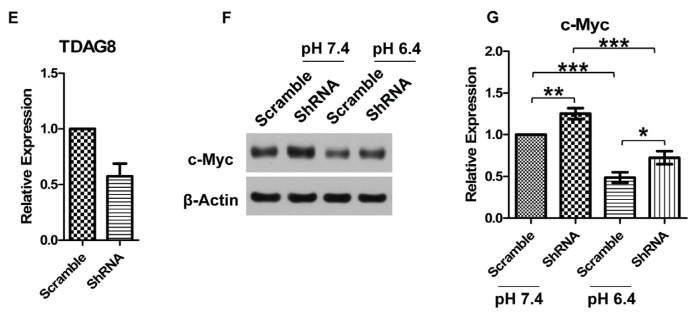
The expression of c-Myc protein is decreased by TDAG8 overexpression and increased by TDAG8 knockdown in U937 cells. (**A**) TDAG8 is highly expressed in U937 cells. Total RNA was isolated from U937 cells and subject to RT-PCR using gene-specific primers of GPR4, G2A, TDAG8 and OGR1. β-Actin was used as a positive control; (**B**) Real-time RT-PCR detecting TDAG8 expression in U937/vector and U937/TDAG8 cells; (**C**) U937/vector and U937/TDAG8 cells were treated with HEM-buffered RPMI (Roswell Park Memorial Institute) medium at pH7.4 or pH 6.4 for 3 h and then subject to Western blot using anti-c-Myc antibody. β-Actin was used as a loading control; (**D**) Quantification of Western blot results. The expression of c-Myc in U937/vector cells under the three-hour pH 7.4 treatment was set as one; (**E**) Real-time RT-PCR of TDAG8 in U937/scramble-shRNA and U937/TDAG8-shRNA cells; (**F**) U937/scramble-shRNA and U937/TDAG8-shRNA cells were treated with HEM-buffered RPMI medium at pH7.4 or pH 6.4 for 3 h and, then, subject to Western blot using anti-c-Myc antibody. β-Actin was used as a loading control; (**G**) Quantification of Western blot results. The expression of c-Myc in U937/scramble-shRNA cells under the pH 7.4 treatment was set as one. The quantification of Western blot data was derived from at least five biological replicates. Error bars indicate ±SEM. *p < 0.05; **p < 0.01; ***p < 0.001.

We next investigated the molecular mechanisms by which U937 lymphoma cells sense the acidic microenvironment and cause c-Myc downregulation. Previous studies demonstrate that TDAG8 and G2A are predominantly expressed in lymphoid tissues and leukocytes [43–47,50]. Our RT-PCR results also showed that among the proton-sensing GPCRs, TDAG8 and G2A have the highest expression levels and OGR1 has a relatively low expression, whereas GPR4 is barely detectable in U937 lymphoma cells (Figure 3A). Because it has been reported that TDAG8 is the major proton sensor in lymphocytes and the proton-sensing function of G2A is controversial [39], we focused on TDAG8 to test whether it is involved in acidosis-induced c-Myc downregulation.

U937 cells were stably transduced with the MSCV (Murine Stem Cell Virus)-IRES (Internal Ribosome Entry Site)-GFP (Green Fluorescent Protein) vector (U937/vector cells) or the MSCV-TDAG8-IRES-GFP construct (U937/TDAG8 cells). Real-time RT-PCR confirmed the overexpression of TDAG8 in U937/TDAG8 cells with a 25-fold increase in mRNA level (Figure 3B). We were not able to examine TDAG8 protein level, because several commercially available TDAG8 antibodies we tested did not work (data not shown). When U937/vector and U937/TDAG8 cells were treated with pH 7.4 and pH 6.4 for 3 h, c-Myc protein was reduced by approximately 50% at pH 6.4 in U937/vector cells (Figure 3C,D), similar to what was observed in parental U937 cells (Figure 1). TDAG8 overexpression further reduced c-Myc expression in the pH 7.4 treatment (24% of decrease) and the pH 6.4 treatment (75% of decrease) when compared to U937/vector cells at pH 7.4 (Figure 3C,D). The partial pH-sensing activity of TDAG8 at pH 7.4 is consistent with previous reports [35,41]. Furthermore, TDAG8 was knocked down by shRNA in U937 cells. The mRNA of TDAG8 was reduced by 50% in U937/TDAG8-shRNA cells in comparison to U937/scramble-shRNA cells (Figure 3E). c-Myc protein expression was increased in U937/TDAG8-shRNA cells when compared to U937/scramble-shRNA cells (Figure 3F,G), which demonstrated that TDAG8 knockdown partially rescued c-Myc expression. This partial rescue may be attributed to these reasons: the knockdown efficiency of TDAG8 is only 50%, and the related family members G2A and OGR1 expressed in U937 cells may cause genetic redundancy. Nonetheless, the results suggest that TDAG8 negatively regulates c-Myc expression in U937 lymphoma cells.

### 2.4. TDAG8 Downregulates c-Myc Protein in a pH-Dependent Manner

**Figure 4:**
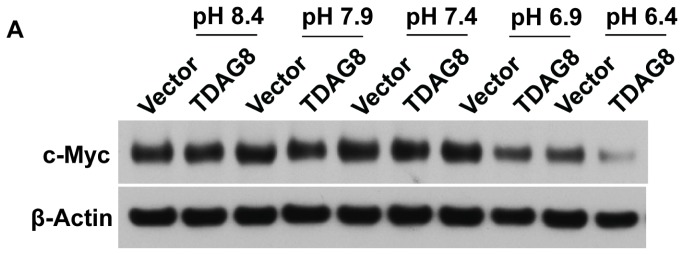

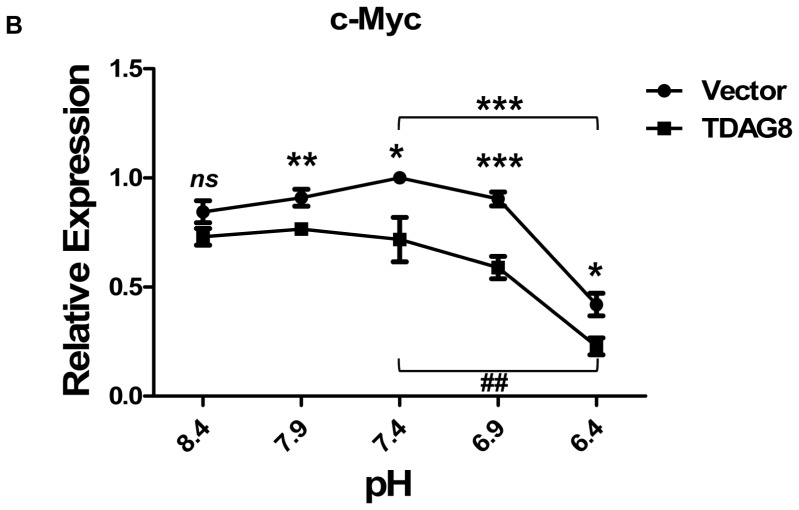
TDAG8 induced c-Myc protein downregulation is pH dependent. (**A**) Cells treated with HEM-buffered RPMI medium at varying pH for 3 h were subject to Western blot assay using anti-c-Myc antibody. β-Actin was used as a loading control; (**B**) Quantification of Western blot results. The expression of c-Myc in U937/vector cells under the 3-h pH 7.4 treatment was set as one. Error bars indicate ±SEM. *p < 0.05; **p < 0.01; ***p < 0.001; ns, not significant (*p* > 0.05), comparing U937/TDAG8 cells to U937/vector cells at each pH, or comparing pH 6.4 to pH 7.4 in U937/vector cells. ## *p* < 0.01, comparing pH 6.4 to pH 7.4 in U937/TDAG8 cells.

We further examined the pH-dependence of c-Myc regulation more thoroughly by assessing the effects of a wide range of pH, including pH 8.4 (4 nM H^+^), pH 7.9 (12.6 nM H^+^), pH 7.4 (40 nM H^+^), pH 6.9 (126 nM H^+^) and pH 6.4 (400 nM H^+^). A left shift of the pH response curve was clearly observed in U937/TDAG8 cells in comparison to U937/vector cells (Figure 4), suggesting that TDAG8 overexpression makes U937 cells more sensitive to the increase of acidity and, also, further reduces c-Myc expression. At pH 8.4 in which TDAG8 has minimal activity, the expression of c-Myc was not significantly different in U937/vector and U937/TDAG8 cells (Figure 4A,B). These results suggest that TDAG8, responsive to the increase of acidity, downregulates c-Myc expression in U937 lymphoma cells.

### 2.5. The Protein Level of c-Myc Is Moderately Increased by Acidosis in H1299 Lung Cancer Cells, but This Increase Is Completely Inhibited by Ectopic TDAG8 Overexpression

**Figure 5:**
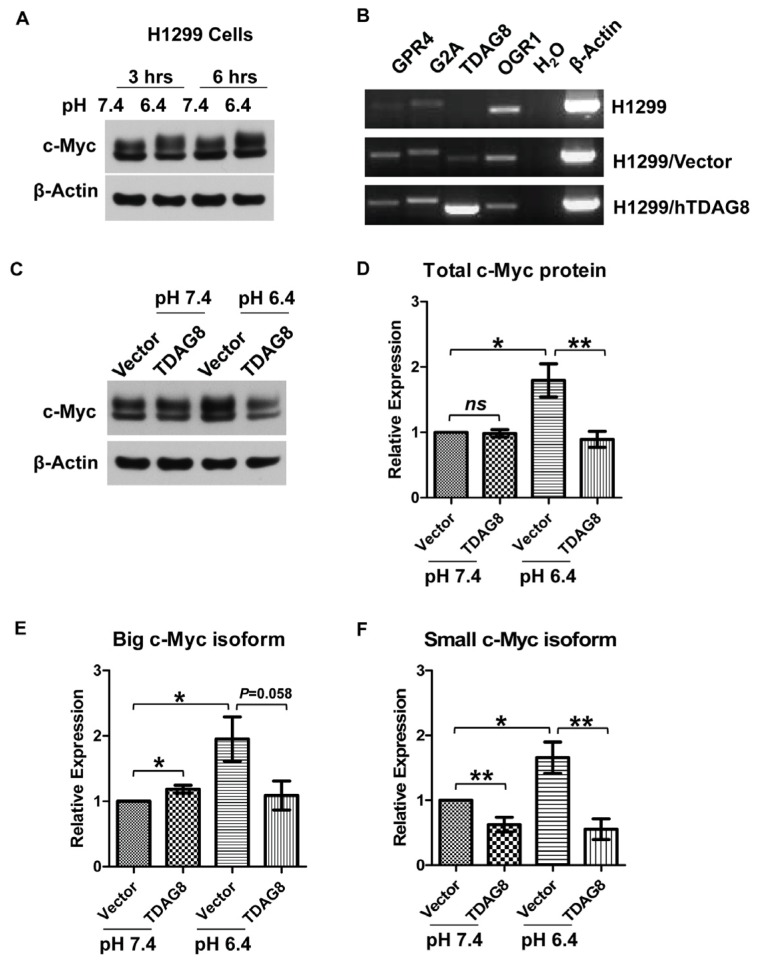
Ectopic overexpression of TDAG8 inhibits the upregulation of c-Myc protein by acidosis in H1299 cells. (**A**) c-Myc protein is moderately increased by acidosis in H1299 cells. Cells treated with HEM-buffered RPMI medium at pH 7.4 or pH 6.4 for three and 6 h were subject to Western blot assay using anti-c-Myc antibody. β-Actin was used as a loading control; (**B**) Low expression of proton-sensing GPCRs in H1299 cells. Total RNAs were isolated from H1299 parental, H1299/vector and H1299/hTDAG8 cells and subject to RT-PCR using gene-specific primers of GPR4, G2A, TDAG8 and OGR1. β-Actin was used as a positive control; (**C**) c-Myc protein is reduced by ectopic overexpression of TDAG8. Cells were treated with HEM-buffered RPMI medium at pH 7.4 or pH 6.4 for 3 h and, then, subject to Western blot assay using anti-c-Myc antibody. β-Actin was used as a loading control; (**D**–**F**) Quantification of Western blot results from (**C**). The expression of the total (**D**), big isoform (**E**) and small isoform (**F**) of c-Myc were quantified based on six Western blot repeats. The expression of c-Myc under the 3-h pH 7.4 treatment was set as one. Error bars indicate ±SEM. *p < 0.05; **p < 0.01; ns, not significant (*p* > 0.05).

To further elucidate the connection between TDAG8 and acidosis-induced c-Myc downregulation, we identified a human lung cancer cell line, H1299, in which c-Myc expression was not decreased by acidic pH (Figure 5A). Western blotting revealed two isoforms of c-Myc protein around 59 and 62 kDa in H1299 cells (Figure 5A). The treatment with pH 6.4, in comparison to pH 7.4, did not result in any reduction of c-Myc protein. Instead, c-Myc protein, particularly the big isoform, was moderately increased by acidosis (Figure 5A). In addition to H1299 cells, we also found that acidic pH did not affect c-Myc protein level in A375 human melanoma cells (data not shown). RT-PCR revealed that the expression of the proton-sensing GPCRs, TDAG8 in particular, was very low in H1299 lung cancer cells (Figure 5B). To test whether ectopic overexpression of TDAG8 can inhibit c-Myc expression, H1299 cells were stably transduced with MSCV-TDAG8-IRES-GFP (H1299/TDAG8 cells) or MSCV-IRES-GFP (H1299/vector cells). The overexpression of TDAG8 in H1299/TDAG8 cells was confirmed by RT-PCR (Figure 5B). As observed in parental H1299 cells (Figure 5A), treatment with pH 6.4 slightly increased the expression of c-Myc protein, especially the big isoform, in H1299/vector cells, whereas ectopic overexpression of TDAG8 completely suppressed the increase of c-Myc by pH 6.4 in H1299/TDAG8 cells (Figure 5C–F).

Collectively, these results demonstrate that acidosis has differential effects on c-Myc expression in different cell types. In U937 lymphoma cells with a high level of TDAG8 and related pH-sensing GPCRs, acidosis clearly decreases c-Myc expression (Figures 1–4). In H1299 lung cancer cells with a low level of pH-sensing GPCRs, acidosis alone is insufficient to reduce c-Myc expression and, instead, slightly increases c-Myc through currently unknown mechanisms (Figure 5). However, ectopic TDAG8 overexpression can suppress c-Myc expression in H1299 cells at acidic pH (Figure 5).

### 2.6. Expression of TDAG8 Transcripts Is Significantly Reduced in Human Lymphomas

**Figure 6:**
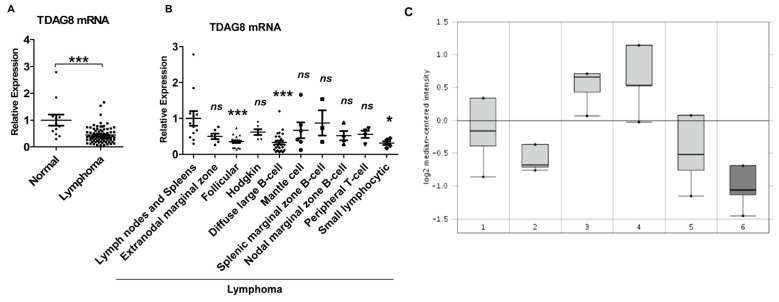
TDAG8 expression is lower in lymphomas than that in normal lymphoid tissues. (**A**,**B**) Human lymphoma tissue cDNA arrays (OriGene Technologies) were subject to real-time RT-PCR using TDAG8 specific primer probes. β-actin was used as an internal control for normalization. TDAG8 expression was compared between non-tumorous lymphoid tissues (lymph nodes and spleens) and all tested lymphoma tissues (**A**) or different subtypes of lymphoma tissues (**B**). The expression of TDAG8 in lymph nodes and spleens was set as one. Each dot represents an individual sample. Error bars indicate ±SEM. *p < 0.05; ***p < 0.001; ns, not significant (*p* > 0.05); (**C**) Analyses of the Oncomine cancer microarray database revealed that TDAG8 expression was reduced by two-fold in follicular lymphoma in comparison to normal lymphocytes (*p* < 0.001). *x*-axis: 1, B-lymphocyte; 2, centroblast; 3, memory B-lymphocyte; 4, naive pregerminal center B-lymphocyte; 5, small cleaved follicle center cell; 6, follicular lymphoma. *y*-axis: log_2_ median-centered intensity.

To correlate the biochemical function of TDAG8 with tumor biology, we compared the expression of TDAG8 transcripts between lymphomas and lymphoid tissues. Real-time RT-PCR was performed on lymphoma tissue cDNA arrays containing 84 cDNA samples from lymphoma patients and 12 cDNA samples from non-tumorous lymph nodes and spleens (see Table S1 for detailed information). The results demonstrated that TDAG8 mRNA expression was decreased by 56% in lymphoma samples in comparison to lymph nodes and spleens (*p* < 0.001) (Figure 6A). To further analyze lymphoma subtypes, the TDAG8 mRNA level was decreased by 60%–70% in follicular lymphoma, diffuse large B-cell lymphoma and small lymphocytic lymphoma samples when compared to normal lymphoid tissues (*p* < 0.05) (Figure 6B). The expression of TDAG8 mRNA was also decreased in other types of lymphomas, such as extranodal marginal zone B-cell lymphoma, Hodgkin’s lymphoma, mantle cell lymphoma, splenic marginal zone B-cell lymphoma, nodal marginal zone B-cell lymphoma and peripheral T-cell lymphoma, but the results were not statistically significant, likely due to the small sample size (Figure 6B). Furthermore, bioinformatic analysis of the Oncomine microarray database revealed that the expression of TDAG8 (GPR65) was significantly lower (2.021 fold decrease, *p* < 0.001) in follicular lymphoma when compared to normal lymphocytes (Figure 6C), concordant with the real-time RT-PCR results of lymphoma samples (Figure 6A,B).

### 2.7. The Level of TDAG8 mRNA Is Downregulated by Acidosis in U937 Lymphoma Cells

**Figure 7:**
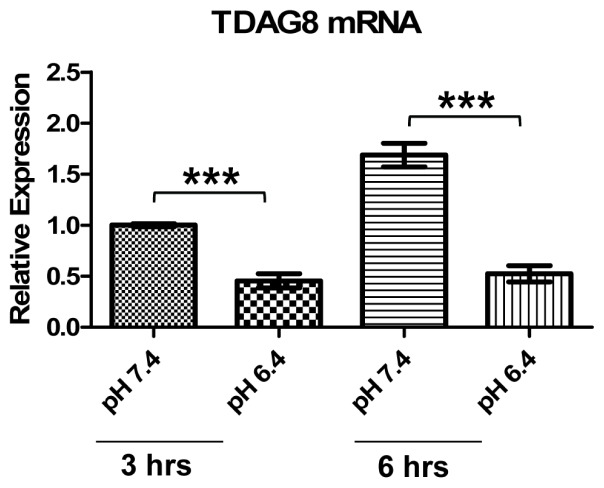
TDAG8 mRNA is decreased by acidosis in U937 cells. U937 cells treated with pH 7.4 or pH 6.4 for three and 6 h were subject to total RNA isolation and real-time RT-PCR using TDAG8-specific primer probes. β-actin was used as an internal control for normalization. TDAG8 expression under the 3-h pH 7.4 treatment was set as one. The data were derived from three biological replicates. Error bars indicate ±SEM. ***p < 0.001.

Since the tumor microenvironment is characteristically acidic, we investigated whether acidosis might affect TDAG8 expression in U937 lymphoma cells. The real-time RT-PCR results showed that TDAG8 mRNA was reduced by more than 50% under the three-hour pH 6.4 treatment compared to the pH 7.4 treatment (Figure 7). When the treatment was extended to 6 h, the reduction of TDAG8 mRNA by acidic pH was about 70%, whereas the TDAG8 level was moderately increased in the pH 7.4 treatment group (Figure 7). These results suggest that acidosis can downregulate the expression of the TDAG8 pH-sensing receptor, and this may represent a potential mechanism by which TDAG8 mRNA is decreased in lymphomas in comparison to non-tumorous lymphoid tissues, due to acidosis in the tumor microenvironment (Figure 6).

## 3. Discussion

Acidosis, a hallmark of the tumor microenvironment, results from excessive proton production and accumulation in a tumor. Initially observed by Otto Warburg decades ago, cancer cells preferentially utilize glycolysis, instead of oxidative phosphorylation, to generate ATP and other molecules, even in the presence of oxygen [20]. Furthermore, the tumor microenvironment is often hypoxic, due to abnormal tumor blood vessels and defective blood perfusion [30,51]. Consequently, glucose uptake is increased in cancer cells as an adaptation mechanism, and a large amount of lactic acid is produced through glycolysis. The Warburg effect is particularly prominent in lymphoma patients as demonstrated by clinical observations that most lymphomas have high activity in ^18^F-fludeoxyglucose-positron emission tomography (FDG-PET) [52,53]. Some lymphoma patients even develop systemic acidosis, because of the large tumor burden and excessive lactic acid production, which is associated with a high mortality rate and poor prognosis [54,55]. Thus, it is important to understand how acidosis regulates the cellular and molecular changes of cancer cells.

In this study, we demonstrate that acute acidosis treatment decreases the expression of the c-Myc oncogene in U937 lymphoma cells (Figures 1–4). It has been reported that acute acidosis can cause cell apoptosis and necrosis, inhibit cell proliferation and delay carcinogenesis [22–24,26,56]. Episodic, transient acidosis is proposed to be a possible mechanism for cancer prevention related to increased physical activity [24]. On the other hand, acidosis can facilitate chromosomal instability, gene mutation, extracellular matrix degradation and tumor invasion [28–30,33,34,57]. Whereas acute acidosis suppresses cell proliferation, chronic exposure to acidosis selects for resistant cancer cells in which autophagy is increased to promote cell survival [31,32]. Because c-Myc is a key regulator of the cell cycle, it will be of interest to examine whether c-Myc downregulation by acute acidosis is essential for inhibiting cell proliferation and how chronic acidosis may affect c-Myc expression in cancer cells.

The expression of c-Myc is tightly regulated by transcription, mRNA stability, translation and protein modification and stability [5,16]. Our results show that the stability of c-Myc mRNA and protein is not affected by acidic pH in U937 lymphoma cells (Figure 2B,C). Instead, acidosis treatment decreases the level of c-Myc transcripts by approximately 50%, which is consistent with the level of c-Myc protein reduction by acidosis (Figures 1 and 2A). These data indicate that acidosis inhibits the transcription of the c-Myc gene. It has been shown that c-Myc gene transcription can be regulated by many transcription factors, chromatin modulators and signaling pathways, such as TCF, FBP, SMAD, LEF-1, β-catenin, γ-Catenin, E2F, AP1, Sp1-3, p53, NF-κB, BRD4, PI3K-Akt-mTOR and Ras-Raf-MEK-ERK/MAPK [17]. The detailed mechanism by which acidosis decreases c-Myc transcription remains to be elucidated.

Our data reveal that the proton-sensing receptor, TDAG8 (GPR65), is involved in acidosis-induced c-Myc downregulation in U937 cells. TDAG8 is a member of the proton-sensing GPCR family, which is activated by acidic extracellular pH through protonation of histidine residues [36,37]. By genetic manipulation of TDAG8 expression, we show that TDAG8, responsive to pH changes, inhibits c-Myc expression in U937 lymphoma cells (Figures 3,4). To the best of our knowledge, this is the first study to demonstrate that acidosis and the pH-sensing TDAG8 receptor regulate c-Myc expression in cancer cells. Interestingly, our quantitative RT-PCR results show that TDAG8 mRNA expression is decreased by more than 50% in human lymphoma samples in comparison to normal lymphoid tissues (Figure 6A,B). Moreover, analyses of the Oncomine cancer microarray database further demonstrate that TDAG8 expression is reduced by two-fold in follicular lymphoma when compared to normal human lymphocytes (Figure 6C). Biochemically, our results reveal that acidosis can downregulate TDAG8 expression in U937 lymphoma cells (Figure 7). This may explain the reduced mRNA level of TDAG8 in human lymphoma samples (Figure 6), because acidosis in the tumor microenvironment may lead to the decrease of TDAG8 expression in lymphoma cells. The downregulation of TDAG8 in lymphomas is different from a previous report that TDAG8 mRNA is overexpressed in a certain percent of kidney, ovarian, colon and breast tumors [58]. However, it should be noted that the quantification of TDAG8 mRNA in that previous study was done by real-time RT-PCR using RNA samples isolated from whole tumor tissues [58]. Because tumors contain many other types of cells, such as infiltrated immune cells, vascular cells and fibroblasts in addition to cancer cells [59], it is unclear if the overexpression of TDAG8 mRNA in these epithelial tumors is directly derived from cancer cells or from infiltrated immune cells, which are known to highly express TDAG8 [43–47,60]. Further studies using *in situ* detection methods and tissue micro-dissection are certainly needed to clarify this issue. Whereas both the pro- and anti-tumorigenic effects of TDAG have been reported in different experimental models and cancer cell types [45,48,49,58], the downregulation of TDAG8 in lymphomas, together with the TDAG8-induced inhibition of c-Myc oncogene expression, suggests that TDAG8 may function as a tumor suppressor in lymphoma.

Because of the critical roles of Myc in tumor biology approaches inhibiting Myc expression, Myc/Max dimerization, Myc/Max DNA binding and Myc target genes have been evaluated for cancer therapy [1]. For instance, compound 10058-F4 inhibits c-Myc/Max heterodimerization and causes apoptosis, cell cycle blockade and differentiation in cancer cells [61–63]. Recently, a small-molecule bromodomain inhibitor, JQ1, has been identified to suppress c-Myc expression and tumor growth through inhibiting the BET bromodomain proteins [64–66]. Despite this progress, no Myc-targeting agents have yet been approved for cancer therapy in the clinic. Therefore, it is of great interest to develop novel approaches to inhibit Myc for cancer treatment and chemoprevention. TDAG8 and related proton-sensing GPCRs may represent potential therapeutic targets. Activation of the TDAG8 receptor, such as by its agonists, may be devised to suppress Myc expression in lymphoma cells. Importantly, GPCRs have traditionally served as important pharmaceutical targets, accounting for 30 to 50 percent of the marketed drugs [67]. A previous study identified a putative TDAG8 agonist BTB09089 [68]; however, the authors state that the agonistic activity of this compound is not strong enough for *in vivo* studies. We have tested the BTB09089 compound in lymphoma cells and found that its efficiency in activating TDAG8 was very limited, if detectable at all [69]. Further research to identify more effective TDAG8 agonists is highly needed.

In conclusion, our results in this study demonstrate that acidosis and the proton-sensing TDAG8 receptor inhibit the expression of c-Myc oncogene in lymphoma cells. Moreover, the expression of TDAG8 is significantly decreased in human lymphomas in comparison to normal lymphoid tissues and lymphocytes, suggesting a potential tumor suppressor role of TDAG8 in lymphoma. These results provide novel insights into the molecular mechanisms by which acidosis in the tumor microenvironment regulates Myc expression and indicate that the proton-sensing GPCRs, such as TDAG8, may be exploited as potential targets for cancer treatment and chemoprevention.

## 4. Experimental Section

### 4.1. Cell Culture and Treatment

U937 human lymphoma cells, Ramos human lymphoma cells, Jurkat human T-cell leukemia cells and H1299 human lung cancer cells (ATCC) were grown in RPMI 1640 medium (Gibco) containing 10% fetal bovine serum in a humidified incubator with 5% CO_2_ at 37 °C. For pH treatment, cells were incubated in RPMI medium buffered with 7.5 mM HEPES (4-(2-hydroxyethyl)-1- piperazineethanesulfonic acid), 7.5 mM EPPS (3-[4-(2-Hydroxyethyl)-1-piperazinyl]propanesulfonic acid) and 7.5 mM MES (2-(*N*-morpholino)ethanesulfonic acid) (collectively known as HEM) at varying pH for 3 h or 6 h. The pH of HEM-buffered medium was adjusted using HCl or NaOH, as previously described [42,70–73]. For the c-Myc protein turnover assay, cells were grown in HEM-buffered RPMI medium (pH 7.4 or pH 6.4) with 50 μg/mL cycloheximide (Sigma, Saint Louis, MO, USA) for various time periods as indicated and, then, subject to total protein isolation. For the c-Myc mRNA decay assay, cells were grown in HEM-buffered RPMI medium (pH 7.4 or pH 6.4) with 5 μg/mL actinomycin D (Invitrogen, Carlsbad, CA, USA) for various time periods, and then, total RNA was isolated.

### 4.2. Western Blotting

Cells were lysed with ice-cold RIPA (radioimmunoprecipitation assay) buffer, and Western blotting was performed as previously described [70–72]. Fifteen micrograms of total protein lysate were mixed with LDS (lithium dodecyl sulfate) sample buffer (Invitrogen, Carlsbad, CA, USA) plus 50 mM DTT (dithiothreitol) and incubated at 70 °C for 10 min. Protein samples were then separated by SDS (sodium dodecyl sulfate)-polyacrylamide gel electrophoresis and transferred to nitrocellulose membranes (Invitrogen, Carlsbad, CA, USA). Membranes were blocked in TBST buffer (Tris-buffered saline with 0.1% Tween-20) containing 5% BSA (bovine serum albumin) overnight and, then, incubated in the same blocking buffer plus anti-c-Myc antibody (D84C12, Cell Signaling, Beverly, CA, USA). After being washed with TBST buffer 3 times, membranes were further incubated with horseradish peroxidase (HRP)-conjugated goat anti-mouse IgG secondary antibody (Cell Signaling), and protein bands were detected using the Amersham ECL (enhanced chemiluminescence) Select Western blotting detection kit (GE Healthcare, Piscataway, NJ, USA). Anti-β-actin primary antibody (Cell Signaling) was used for the loading control. Western blot results were quantified using the LabWorks software (UVP BioImaging Systems, Upland, CA, USA), and the c-Myc protein level was normalized by β-actin loading control.

### 4.3. RNA Extraction, RT-PCR (Reverse Transcriptase-Polymerase Chain Reaction) and Quantitative Real-Time RT-PCR

Total RNA was isolated using the RNeasy Plus kit (QIAGEN, Valencia, CA, USA). cDNA was synthesized using SuperScript II reverse transcriptase (Invitrogen, Carlsbad, USA). Regular RT-PCR was performed using GPR4-, G2A-, TDAG8-, OGR1- and β-Actin-specific primers with a program of 95 °C for 2 min, followed by 35 cycles of 94 °C for 30 s, 58 °C for 30 s and 72 °C for 1 min. The primer sequences are: GPR4 5′-GCTCCCAGATCCCATCACAG-3′ and 5′-CACTTTCCCCCGAGTCACAG-3′, OGR1 5′-AACAGGGTGGGAAGGGAAGA-3′ and 5′-TGGGCCTCCCTTTGGTAGAT-3′, G2A 5′-TCACCAACCACCGGATTTTC-3′ and 5′-GGCTCAGCAGGACTCCTCAA-3′, TDAG8 5′-GCATTGCCGTTGATCGGTAT-3′ and 5′-AATGCAGCGAATCAGCAACA-3′, and β-Actin 5′-TGACCCAGATCATGTTTGAGA-3′ and 5′-AGGTCCAGACGCAGGATG-3′. Real-time TaqMan PCR was performed using the pre-designed TaqMan primer-probes (Invitrogen) for c-Myc (catalog # Hs00153408_m1), TDAG8 (catalog # Hs00269247_s1), GAPDH (catalog # Hs99999905_m1) and 18S rRNA (catalog # Hs99999901_s1), as previously described [71,72]. Lymphoma tissue cDNA arrays were purchased from OriGene Technologies (catalog # LYRT301 and LYRT302) and subject to real-time PCR using specific primer-probes for TDAG8 and β-Actin (catalog # Hs99999903_m1). The tissue cDNA arrays contained 84 lymphoma samples from patients with various types of lymphoma and 12 control lymph nodes and spleens without any tumor (for detailed information, see Table S1 and the vendor’s website: http://www.origene.com/qPCR/Tissue-qPCR-Arrays.aspx).

### 4.4. Plasmid Constructs

The open reading frame of human TDAG8 (GenBank accession # NM_003608) was amplified using the primers designed to contain EcoRI and NotI sites: 5′-ATAAGAATGAATTCACCATGAACAGCACATGTATTGAAGAA-3′ and 5′-ATAAGAATGC GGCCGCCTACTCAAGGACCTCTAATTCCAT-3′. The PCR product was digested with EcoRI and NotI and cloned into the retroviral expression vector MSCV-IRES-GFP to generate the MSCV-TDAG8-IRES-GFP construct. The TDAG8 shRNA construct pLKO.1-TRCN0000011645 (RHS4533-NM_003608) was purchased from Thermo Scientific, and the control scramble shRNA construct was purchased from Addgene (plasmid 1864).

### 4.5. Cell Transfection and Transduction

To generate a stable TDAG8-overexpressing cell line, retrovirus-mediated cell transduction was performed as previously described [74]. Briefly, the MSCV-TDAG8-IRES-GFP construct was co-transfected with the pCL10A-1 packaging vector into HEK 293T cells to produce retroviral particles, which were used for cell transduction to generate U937/TDAG8 and H1299/TDAG8 cells. MSCV-IRES-GFP virus was used to generate the control cell lines, U937/vector and H1299/vector cells. Lentivirus-based cell transduction was performed to generate the TDAD8 knockdown cell line. pLKO.1-TRCN0000011645 was co-transfected with the envelope plasmid pCMV-VSV-G (Addgene, plasmid 8454) and packaging plasmid pCMV-dR8.2 dvpr (Addgene, plasmid 8455) into HEK 293T cells to produce lentiviral particles, which were then used for cell transduction to generate U937/TDAG8-shRNA cells. Cells with stable exogenous gene expression were selected by 2 μg/mL puromycin. A scramble shRNA lentiviral vector was used to generate the control U937/scramble-shRNA cells.

### 4.6. Oncomine Database Mining

Bioinformatic analysis was performed by mining the Oncomine cancer microarray database [75]. The gene expression of TDAG8 (GPR65) was compared between normal lymphocytes and follicular lymphoma samples (data retrieved July, 2013).

### 4.7. Statistical Analysis

Data were subject to statistical analysis using the GraphPad Prism 5 software (GraphPad Software, Inc., La Jolla, CA, USA). Each statistical analysis was derived from at least three independent biological replicates. Statistical significance was determined using the unpaired *t*-test. *p* < 0.05 is considered statistically significant.

## 5. Conclusions

Acidosis and the pH-sensing receptor, TDAG8, inhibit c-Myc oncogene expression in human lymphoma cells; TDAG8 transcripts are significantly decreased in human lymphomas in comparison to non-tumorous lymphoid tissues.
